# Syphilis and Its Relation to the Oral Cavity

**Published:** 1914-05-15

**Authors:** Harry F. Parks


### SYPHILIS AND ITS RELATION TO THE ORAL CAVITY.

BY HARRY F. PARKS, D.D.S.

Read before the Jackson Dental Club, Feb., 1914.

*"Keep your hands from literary picking and stealing: But if you cannot refrain from this kind of stealth, abstain from murdering what you steal."—Toplady.*

In treating a subject of this nature one must of necessity resort to more or less "literary picking," and I plead guilty to this, but I have at least, attempted to minimize the number of my murders.

The past decade has seen so many wonderful developments in Dental Science that even the Medical Profession, which in years gone by, has looked upon us in pity and scorn and unworthy to sit in the councils of the mighty (?) are according us some of the credit long due.

We are constantly hearing of the wonderful development in the technical and clinical departments of our profession. Even our manufacturers are putting forth untiring efforts to meet the constantly increasing demands of the profession, and have accomplished wonders in the matter of filling materials that not only possess tooth saving qualities but far surpass in esthetic effect anything heretofore known. This is good and we all rejoice in it. But not among the least of the great strides we are making is the work of our Pathologists and Therapeutists. Startling revelations have been made in regard to the Oral Cavity as a bacteriological breeding ground or culture medium. Many mouth organisms have been isolated and studied, and the knowledge thus gained has very materially assisted us in clearing up some vexing problems and aided us in avoiding many unpleasant pitfalls.

But the object of the essayist is not to enumerate or comment on this large number of organisms found in the mouth, but to call attention again to one which, owing to its virulence, together with the difficulty of its detection, becomes the most dangerous and far reaching of them all, namely; Spyrochete Pallida, producing Syphlis. This disease is so universally prevalent and its signs are so frequently exhibited in the mouth, and there are such great chances for innocently transferring from one patient to another that it is necessary that the dentist should know how to detect it. Dental Surgery always was and always will be, very largely mechanical, and without mechanical instinct, the would-be Dental Surgeon is fordoomed to failure, but the time has come for a broader knowledge of disease conditions and is imperative.

To practice the Science—for it is a Science—in its truest and highest conception—we must go farther than the art and mechanical practices. We must be preventive in our methods to be up to date. Preventive not only in originating and controlling, but in spreading of diseases from patient to patients of those diseases that so commonly affect the Oral Cavity.

Syphilis as described by Osler, is a specific disease, of slow evolution, caused by the Spyrochete pallida propagated by inoculation (acquired Syphilis), or by heriditary transmission (congenital syphilis).

Many things can be said of the different stages in its development, all of great interest, in a general way, but we shall not discuss them at this time, as your essayist wishes to confine the paper to Oral Syphilis only as it practically interests the dentist.

As dentists we are interested chiefly in labial, lingual and Tonsilar infection and manifestations. Strange as it may seem, statistics show the lips to be the most common avenue of infection of extra genital Syphilis. Possibly because the lip membranes are more exposed and often in condition to receive inoculation.

Out of 727 cases examined, 567 presented lip chancre;

75 on the tongue, tonsil 69, gums 11, soft palate and pillars 4. The internal aspect of the cheek 1.

In defining chancre, I cannot do better than quote Volume III of Kingsbury's Dermochromes. Chancre is the initial lesion of acquired syphilis; and develops at the point of contagion. It is of neoplastic growth seated on cutáneous or mucus surface and presents two general types. Lesion with a flat surface and underlying indurations, or an indurated nodular mass varying in size with the age of lesion. A labial chancre usually presents itself as a solitary lesion and is most common to lower lip. It is situated on cutaneous or mucus surface although both may be involved. The mucus patch bears a close resemblance to a canker sore but the induration is very pronounced and lip is everted.

A cutaneous lesion may become ulcerated with deep excavations and bears close resemblance to a malignant growth but is differentiated by its rapid evolution. Chancre of the tongue is round and oval and is usually found on superior surface of the anterior third. Has the general appearance of erosion but may present itself in saucer shape with marked indurations. Chancre of the tonsil presents three types that will not be discussed here as they are not of special interest to the Dental Surgeon owing to the infrequency of their appearance in his practice. Such lesions are however very virile and can be easily transmitted unless great care is taken.

In regard to congenital syphilis in its relation to the Oral Cavity, literature is strangely silent, except when it has produced malformed teeth or jaws, or for its secondary influences.

We have very little interest in this as it is rarely if ever that this type is transmissable extra genitally. There are many ways of conveying syphilitic infection from the mouth. It is a lamentable clinical fact that sexual perversion is one of these, but beyond this, recognition, need not be discussed. Undoubtedly, a large number of these cases are due to ignorance.

Promiscuous kissing, water glasses, pipes and various other methods. Carelessness in the matter of instruments of both Surgeons and Dentists may become actively instrumental in spreading of this disease among the innocent.

It is for the purpose of calling attention to the last named means that I present this paper, hoping for good discussions of practicable means of prevention.

While "Ignorance of the law excuses no man," carelessness of the law, whose penalty he well knows is even less excusable. We may not be able always to recognize oral syphilis, but we should be watchful and careful of our operations at all times.

We, as Dental Surgeons, must face the fact that any transmissions of this disease by the medium of our instruments is due to carelessness. We cannot even fall back on the plea of ignorance. We have had sufficient instruction in this matter to give us a working basis, and while it is many times impossible to diagnose the case with any degree of accuracy, we can see sufficient evidence to give us warning—warning which we can ill afford to ignore. When we see these signals, or have our suspicion aroused, we should handle the case in the same manner we would a positive diagnosis. It is better far to be safe than sorry, and the feeling of security which it gives is ample remuneration for the extra precaution taken. Ordinary cleanliness will not suffice at such times. We must take extra precautions in the matter of sterilization.

Everything that comes in contact with the mouth or instruments used should be thoroughly boiled. Rubber gloves should be used and subjected to the same treatment. This is but a repetition of the old story, but it will not harm us to review it again. We are all apt to become weary in well doing, but success in this, as in everything else worth while, is to be had only at the price of eternal vigilance.

In the wave of Health Hygiene that is sweeping over the country, the Dental Surgeon seems to be taking no small part. His daily routine commits to his care one of the highly important parts of the Human Anatomy. It is the gateway to the entire physical organization, and as such should be well guarded. Our personal safety, as well as the safety of our patients demands extraordinary precaution in all as well as suspected cases. It is expected of us as guardians of the mouth, and it will be a blessed heritage if we can measure up to the obligation.

It is our birth-right handed down by those sturdy pioneers of Dentistry, those grand old men, whose face to the front attitude has made possible the wonderful achievements of to-day in our profession. Let us be up and doing and be worthy of the trust and above all, let us not sell our birth-right for a mess of pottage, as some with the low horizon and near view seem determined to compel us all to practice.

As Dr. Charles Mayo has put it, "the health of the future generations depends upon you as Dental Surgeons." "Will you be equal to it?"

We shall be if we can arise to our opportunity, but we may not if we neglect to make ourselves intelligent on every pathologic condition that comes within our province or fail to handle all such conditions with more than ordinary precautions.

